# Evidence for perennial malaria in rural and urban areas under the Sudanian climate of Kandi, Northeastern Benin

**DOI:** 10.1186/1756-3305-7-79

**Published:** 2014-02-24

**Authors:** Renaud Govoetchan, Virgile Gnanguenon, Roseric Azondékon, Rodrigue Fiacre Agossa, Arthur Sovi, Frédéric Oké-Agbo, Razaki Ossè, Martin Akogbéto

**Affiliations:** 1 Centre de Recherche Entomologique de Cotonou (CREC), 06 BP 2604 Cotonou, Bénin; 2 Faculté des Sciences et Techniques, Université d’Abomey Calavi, Calavi, Bénin; 3 University of Massachusetts Amherst, Amherst, United States of America

**Keywords:** Drought, Malaria, Prevalence, Domestic larval habitat, Vulnerability, Children

## Abstract

**Background:**

In arid settings, droughts usually lead to periods of very low or no malaria transmission. However, in rural Kandi (Sonsoro) in northeastern Benin, several malaria cases are often diagnosed during dry seasons. The underlying factors accounting for this phenomenon remain unknown.

**Methods:**

The entomological profile of Sonsoro has been studied compared to a location in urban Kandi (Gansosso) for a period of one year. During this period, *Anopheles* larval habitats were investigated and populations of *Anopheles gambiae s.l.* were sampled by human landing catches in both areas. Enzyme-linked immunosorbent assays (ELISA) for *Plasmodium falciparum* circumsporozoite protein (CSP) were conducted on vector specimens and the entomological inoculation rates (EIR) were determined per season (wet versus dry) in each area. In addition, during the severe drought period, Rapid Diagnostic Tests (RDTs) were conducted on school children under the age 10 years in these areas to provide a global view of drought-malaria prevalence and to perform a crossing with entomological data in Kandi.

**Results:**

Overall, *An. gambiae s.l.* was particularly abundant in rural Kandi compared to the urban area with a significant decrease of vector density in both sites during the dry season. In this period, larval sampling data identified household water sources as potential breeding sites in urban and rural Kandi. We also observed a significant seasonal variation of the infectivity rate in both areas but for each period (season), the EIR was higher in the rural site than in the urban. Data of *P. falciparum* detection was the reflection of entomological findings. The drought-malaria prevalence was 5.5 times higher in rural Kandi as compared to urban Kandi. The presence of a permanent water site and the low level of urbanization in rural Kandi were identified as a risk factor.

**Conclusion:**

Our data showed a high level of malaria transmission in the municipality of Kandi. Household water source plays an important role in maintaining the breeding of anopheles larvae and the malaria transmission in Kandi. In rural settings, the proximity to permanent water sites could probably be the aggravating factor.

## Background

Malaria is a major cause of global morbidity and mortality, with most of the burden in sub-Saharan Africa [1,2]. More than 90% of recorded malarial deaths occurring in Africa are among the most vulnerable population, such as children under five years old and pregnant women [3,4]. In spite of many efforts of the National Malaria Control Programs (NMCP) and international donors to combat the disease through various vector control interventions, malaria continues to be a real public health problem.

In Benin, malaria was the leading reason for consultation and hospitalization in healthcare centers. In 2012, incidence of malaria in Kandi, northeastern Benin, was 28.55%, two times higher than the national average of 14.6 per 100 Benineses [5]. Despite a dry season period of about six months, in the Kandi municipality, several cases of malaria were still diagnosed. Healthcare registers in the health center of Sonsoro (rural Kandi) indicated that during the drought period between 2008 and 2012, clinical malaria cases were estimated at 15 to 30%. Of these, approximately nearly 60% were children under 10 years old (R. Govoetchan, personal communication). Although these unexpected cases could be linked to relapse or cases of imported malaria, the possibility of recent infection could not be ruled out in the light of the magnitude of the prevalence and the sedentary status of local people and especially children.

Several studies have shown that malaria infection is influenced by environmental factors such as temperature, rainfall, humidity and elevation. In tropical settings, temperature and rainfall conditions are nearly always favorable for the development of *Anopheles* mosquitoes, the intermediate hosts in the transmission of malaria parasites [6,7]. According to Martin and Lefebvre [8], rain is generally synonymous with new mosquito breeding sites. However, rain can also destroy the existing ones: heavy rains can transform basins to streams, hinder the development of eggs and larvae, or simply eject them from water [8]. Conversely, extreme drought conditions lead to the evaporation of most of the conventional mosquito breeding sites and in turn lower the mosquito population [8-12]. For this reason, the dry season in tropical Africa is often regarded as a period of very low malaria transmission, a period during which no method of malaria prevention and control is deployed. This is due to the fact that, like most mosquitoes, *Anopheles gambiae*, depends on the availability of suitable surface water for oviposition. However, during the dry season and dry spells during the wet season, larval sites may not be available for days, weeks, or even months in different environments [13-16]. Under such conditions, mosquitoes that develop eggs (gravid specimens) cannot lay them [17] thereby seriously affecting the population size. Thus, the *Culicidae* nuisance and the anopheline density are significantly lowered compared to the wet periods of high transmission.

An important reason for the heavy malaria burden in Africa is that the malaria-transmission machinery consists of an exceptionally robust vectorial system including *An. gambiae* s.s (representing two incipient species known as the M and S molecular forms), a sibling species *An. arabiensis*, and *An. funestus*[18]. These vectors are not only able to exploit diverse environments including expansive dry savannas and semi-desert areas, but also able to ensure the maintenance of malaria transmission during periods of severe drought. Moreover, many studies reported changes in people’s behavior in relation to the individual methods of protection during the dry season. Given that the mosquito bites are less noticeable at this time of the year and that the night is sometimes excessively hot, people do not feel the need to sleep under bed nets and trivialize any other method of protection against possible scarce mosquito bites.

Very few scientific publications focusing on malaria during the dry season exist. In order to assess the sources of the several recent *Plasmodium falciparum* infections often reported during drought periods in Sonsoro, rural Kandi, we decided to conduct a year round study on the entomological profile of the location compared to a site in urban Kandi (Gansosso). We particularly and meticulously focused on what happens in the dry season. Moreover, we investigated malaria prevalence in the two locations (Sonsoro and Gansosso) during a period of aridity, a time supposed to be of very low or zero malaria transmission, and compared them with drought entomological data.

## Methods

### Description of sites

**Figure 1:**
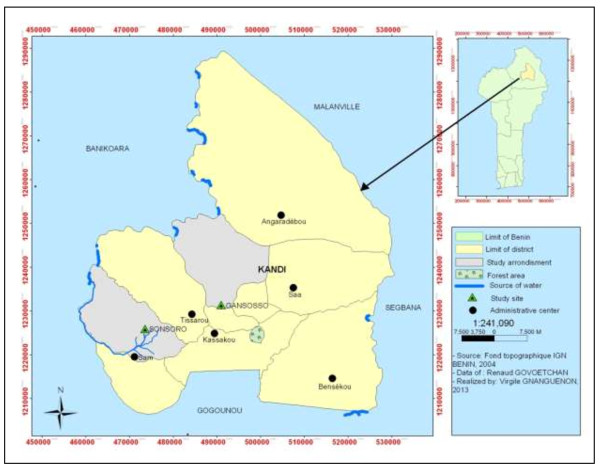
Map of the municipality of Kandi showing the study site in rural and urban area.

This study was carried out in Kandi (11 ° 07 ‘43 "N, 2 ° 56' 13 E), northeast of Benin. Kandi is under a Sudanian climate with a dry season from November to April and a Wet season from May to October. During the dry season, temperatures are very high and up to 45°C can be recorded. The drought is often severe, with a lot of sunshine and can last up to six months. Our data collection was held precisely in Gansosso and Sonsoro. Gansosso is an urban settlement in Kandi with no water site. Sonsoro on the other hand is located in a rural area, 20 km from the city center and is bordered by the Sota tributary, a permanent water site (Figure 1). The persistence of a water site in Sonsoro is a cause of proliferation of mosquitoes in this location (Govoetchan, personal communication).

### Entomological data collection

#### ***Assessing the dry season refugia for anopheline larvae***

##### 

**Identification and geo-positioning of mosquito breeding sites** The data collection was carried out for one year (from May 2012 to April 2013). Samplings were carried out exhaustively every two months during the wet season (May 2012 to October 2012), and each month during the dry season (November 2012 to April 2013). Our investigations became monthly in the dry season to ensure the identification and listing of all aquatic habitats that could potentially serve as mosquito breeding during periods of severe aridity. At each study site, all aquatic habitats were explored using a dipper (60 cm^3^ of volume) and any habitat harboring at least one mosquito larva was identified as a positive breeding site and its nature was recorded and geo-referenced. While the breeding site is registered, a brief taxonomic identification of larvae found was performed through the genera [19].

##### Collection method

The tools used for data collection were tablets Samsung Galaxy Tab® 10.1. An electronic survey form was created for this purpose with the ODK (Open Data Kit) collect software and enabled the automatic recording of data in the field. This survey form allows an instantaneous measurement of larval data for positive breeding sites identified. At the end of each study visit, the data is transferred directly to a cloud server in order to ensure their backup and their traceability.

##### 

***Anopheles* adult collection** The study of the seasonal trend of malaria transmission in each site was performed through *Anopheles* vector collections. We evaluated per season the biting rate and the frequency of infected biting for each study site. Mosquitoes were collected using human landing catch inside and outside of two dwellings. The entomological data collection was carried out from May 2012 to April 2013. Samplings were carried out every two months during the wet season (May 2012 to October 2012), and each month during the dry season (November 2012 to April 2013). Our investigation was done every month during drought for a good assessment of the entomological situation in this period of the year and a proper evaluation of malaria risk. The mosquito captures were made from 9:00 pm to 5:00 am. Two successive human landing catches were carried out per mission for a total of 16 captures per mission for the two study sites.

### Laboratory processing

The collected mosquitoes were identified in the morning into genus and species level using the taxonomic key by Gillies and de Meillon [19]. Anopheline infectivity rates were determined by enzyme-linked immunosorbent assay (ELISA) using monoclonal antibodies against *Plasmodium falciparum* circumsporozoite protein (CSP) [20].

Results from ELISA test and the mosquitoes sampled were used to determine the number of bites per man per night and per season (wet versus dry) for each location, the sporozoitic index and the EIR of *An. gambiae* per season*.*

#### ***Prevalence of malaria during the dry season***

We conducted a cross-sectional survey on the prevalence of malaria during a high dry season (March 2013), among schoolchildren in Sonsoro and Gansosso.

#### ***Enrolment of study patients***

We identified the public primary schools of Sonsoro and Gansosso. In each school, we selected 200 children. Inclusion criteria were age < 10 years (we then started from the lower classes successively kindergarten, followed by first, second and third levels of primary schools), residents in Sonsoro or Gansosso. Parental consent was obtained from parents of children involved in the study.

#### ***Method of diagnosis***

Diagnosis of malaria wasperformed using the two-band CareStart™ Malaria HRP-2 test [21]. It is the RDT currently recommended by Benin NMCP for use in areas where reliable microscopy is not available. It is a lateral flow antigen detection test in a cassette format targeting Histidine-Rich Protein II (HRP-2), produced by trophozoites and young gametocytes of *Plasmodium falciparum*. HRP-2 RDTs have shown good sensitivity in a variety of field settings [22-24] and are reported to be the best diagnostic choice for areas with medium-to-high malaria transmission rates in Africa [25]. The detection threshold is approximately 100 parasites/μl.

#### ***Diagnostic procedures***

Study staff performed RDTs by pricking the finger to obtain peripheral blood samples according to the manufacturers instructions (AccessBio, New Jersey, US). A pipette was used to transfer blood from the finger onto the test. A buffer solution was then applied, which carried the blood up the cellulose nitrate strip, over the test and control lines. The results were interpreted and recorded after 15 to 30 min. Results were recorded as negative, positive or invalid.

#### ***Data analysis***

Larval data were copied from the cloud server and handled using Epi Info (version 6.04; Centers for Disease Control and Prevention). The seasonal frequencies of various types of mosquito breeding sites were calculated and pairwise-compared by the Poisson test to determine significance of results. The EIR, the key entomological parameter representing the number of infected anopheline bites received by one human per unit of time (night, month or year) was calculated for each study site and each season in order to measure the burden of malaria transmission in each period (season) of the year. The EIR values were analyzed through the Poisson test and compared between seasons (wet versus dry) inside a same location and between the two study sites (rural versus urban). The drought-malaria prevalence data were compared between locations (rural versus urban) using Chi^2^ test and their confidence limits determined using the binomial confidence limit method. The Wald test and the LR test were performed to appreciate the significance of the impact of some collateral factors (age, weight, sex, educational level, urbanization) on the frequency of malaria. A p-value < 0.05 was considered as significant. These tests were conducted using R 2.14.1 software.

#### ***Ethical considerations***

Ethical approval for this study was granted by the Ethical Committee of the Ministry of Health in Benin. Community leaders were briefed on the protocol before the study and gave verbal consent before the study began. Written consent was obtained from all participating volunteers, who were vaccinated against yellow fever and provided with malaria prevention and curative treatments according to World Health Organization (WHO) recommended regimen (on the basis of fever and Detectable *P. falciparum* parasitemia). Regarding the cross-sectional survey on the malaria prevalence, parental written consents were obtained from parents of children involved in the study.

## Results

### Entomological profile of the study sites

#### ***Dry season refugia for mosquito larvae***

A total of 126 mosquito larval habitats were identified in Sonsoro (rural) and Gansosso (Urban) between May 2012 and April 2013 of which 30.95% were recorded during the dry season (Table 1). In both sites, the trend is basically the same and mosquito breeding habitats were of a more diversified nature depending on the season. In the wet season (May-October 2012), rainwater collections and various holes were the majority constituting together 51.43% of all larval habitats recorded in rural Kandi and 76.92% in urban Kandi. However, during the long drought that lasts about six months (November 2012-April 2013), these classical mosquito breeding sites disappeared altogether. The drought-refugia for mosquito breeding were mainly the household canisters, jars, flower pots that we defined as domestic larval habitats. Moreover cisterns for water supplies and wells have been identified as typical refugia of mosquito breeding during drought periods.

**Table 1:** Seasonal diversity in larval habitats of mosquitoes in rural (Sonsoro) and urban (Gansosso) Kandifrom May 2012 to April 2013

**Study sites**	**Nature of larval habitats**		**Wet season (May-12 to October-13)**			**Dry season (November-12 to April-13)**			**Total**	
		**Total 1**	**%**	**CI-95%**	**Total 2**	**%**	**CI-95%**	**Total**	**%**	**CI-95%**
**Rural Kandi (Sonsoro)**	Rain water collection	13	37.14^a.b^	[21.47- 55.08]	0	0.00^a^	[00.00- 11.95]	13	20.31^a^	[11.28- 32.22]
	Various holes	5	14.29^b.c^	[04.81- 30.26]	0	0.00^a^	[00.00- 11.95]	5	7.81^b^	[02.53- 17.30]
	In shallow aquifer	1	2.86^c^	[00.07- 14.92]	0	0.00^a^	[00.00- 11.95]	1	1.56^b^	[00.04- 08.40]
	Domestic habitats	15	42.86^b^	[26.32- 60.65]	21	72.41^b^	[34.85- 98.73]	36	56.25^a.c^	[09.90- 81.59]
	Cistern/well	0	0.00^c^	[00.00- 10.00]	2	6.90^a^	[00.85- 22.77]	2	3.13^b^	[00.38- 10.84]
	Around public standpipes	0	0.00^c^	[00.00- 10.00]	2	6.90^a^	[00.85- 22.77]	2	3.13^b^	[00.38- 10.84]
	Polluted water collection	0	0.00^c^	[00.00- 10.00]	1	3.45^a^	[00.09- 17.77]	1	1.56^b^	[00.05- 08.40]
	Others	1	2.86^c^	[00.07- 14.92]	3	10.34^a^	[02.19- 27.35]	4	6.25^b^	[01.73- 15.24]
	Total	35	100	-	29	100.00	-	64	100	-
**Urban Kandi (Gansosso)**	Rain water collection	34	65.38^a^	[50.91- 78.03]	0	0.00^a^	[00.00- 30.85]	34	54.84^a^	[41.68- 67.52]
	Various holes	6	11.54^b^	[04.35- 23.44]	0	0.00^a^	[00.00- 30.85]	6	9.68^b^	[03.63- 19.88]
	Domestic habitats	11	21.15^b.c^	[11.70- 46.42]	0	0.00^a^	[00.00- 30.85]	11	17.74^b.c^	[08.42- 31.01]
	Cistern/well	0	0.00^d^	[00.00- 06.85]	5	50.00^b^	[18.71- 81.29]	5	8.06^b^	[02.67- 17.83]
	Polluted water collection	1	1.92^d^	[00.05- 16.26]	5	50.00^b^	[18.71- 81.29]	6	9.68^b^	[03.63- 19.88]
	Total	52	100	-	10	100	-	62	100	-
	Total (Rural + urban)	**87**	**69.05**	[63.49- 76.62]	**39**	**30.95**	[23.38- 36.51]	**126**	100	

CI: confidence interval.

^a,b,c,d^Values with the same superscript do not differ significantly.

However, the type of mosquito larval habitats sampled in drought depended fundamentally on the nature of the study site. During the dry season, the majority of larval habitats sampled in rural settings were of a domestic nature (72.41%), whereas in urban settings (Gansosso), most of the drought-refugia for mosquito breeding were cisterns and wells (50%).

#### ***Genera of mosquitoes identified in the larval habitats***

The main genera of mosquito that we identified in the larval habitats were *Anopheles*, *Culex* and *Aedes* (Table 2). The breeding sites were either exclusive (only one genus of mosquito in the larval habitat), or mixed (two or three genera of mosquito found in sympatry). The prevailing mosquito genus in the larval habitats depended on the seasons of the year and the nature of study site (rural versus urban). In the rural site (Sonsoro), *Anopheles*-exclusive larval habitats were the most sampled in the wet season (48.57%). In this season, very few larval habitats of non-anopheline mosquitoes were recorded in this site (20%). Conversely in urban locations (Gansosso), there were more non-anopheline breeding sites found during the wet season (48.08%).

**Table 2:** Genera of mosquitoes found in thelarval habitats in rural (Sonsoro) and urban (Gansosso) Kandi from May 2012 to April 2013

**Sites**	**Genus of mosquitoes’ larvae**	**Wet season**		**Dry season**			**Total**
		**(May-12 to October-13)**		**(November-12 to April-13)**			
		**Total 1**	**%**	**Total 2**	**%**	**N**	**%**
**Rural Kandi (Sonsoro)**	*An-*exclusive	17	48,57^a^	2	6,90^a^	19	29,69^a^
	*An*-mixed	11	31,43^a,b^	17	58,62^b^	28	43,75^a^
	Non-anopheline	7	20,00^c^	10	34,48^c^	17	26,56^a,c^
	Total	35	100.00	29	100,00	64	100,00
**Urban Kandi (Gansosso)**	*An-*exclusive	19	36,54^a^	2	20,00^a^	21	33,87^a^
	*An*-mixed	8	15,38^b^	1	10,00^b^	9	14,52^b^
	Non-anopheline	25	48,08^c^	7	70,00^c^	32	51,61^c^
	Total	52	100,00	10	100,00	62	100,00
	Total (Rural + urban)	87	**69.05**	**39**	**30.95**	**126**	100.00

*An-*exclusive: *Anopheles*-exclusive larval habitats.

*An-*mixed: *Anopheles*-mixed larval habitats.

non-anopheline: larval habitats of non-anopheline mosquitoes.

^a,b,c^Values with the same superscript do not differ significantly.

In regard to the dry season, we observed that in both sites, there was a significant decrease in *Anopheles*-exclusive larval habitats and an increase in larval habitats of non-anopheline mosquitoes. During the drought period, *Anopheles* larvae were mainly sampled in mixed breeding sites of mosquitoes, especially in rural Kandi (58.62%).

#### ***Refugia of anopheline larvae in drought period***

A total of 22 anopheline larval habitats (exclusive and mixed) were identified in Sonsoro (rural) and Gansosso (urban) during the dry season (November 2012-April 2013). Overall, most of the drought-refugia of anopheline larvae were of domestic nature (68.42% in rural Kandi, and 66.67% in urban Kandi) (Table 3). These results suggested that the poor conservation of water stocks in households is a potential factor in the maintenance of anopheles breeding and malaria transmission not only in rural settings but also in urban settings. Moreover, in rural settings, anopheline larvae were also sampled in cisterns and wells (25%) around public standpipes (16.70%).

**Table 3:** Habitats in anopheline larvae during drought periods in rural (Sonsoro) and urban (Gansosso) Kandi from May 2012 to April 2013

**Village**		**Various holes**	**Domestic habitat**	**Cistern/well**	**Polluted water collection**	**Around public standpipes**	**Total**
**Rural Kandi (Sonsoro)**	N	1	13	3	0	2	19
	%	8.30%^a^	68.42%^b^	25.00%^c^	0.00%^a^	16.70%^c.d^	100.00%
**Urban Kandi (Gansosso)**	N	0	2	0	1	0	3
	%	0.00%^a^	66.67%^b^	0.00%^a^	33.33%^a^	0.00%^a^	100.00%
**Total**	**N**	**1**	**15**	**3**	**1**	**2**	**22**
	**%**	**4.55%**^**a**^	**68.18%**^**b**^	**13.64%**^**c**^	**4.55%**^**a**^	**9.09**^**c.d**^	**100.00%**

^a,b,c,d^Values with the same superscript do not differ significantly.

#### ***Diversity and geo-variability of mosquito density in rural and urban Kandi***

A total of 966 mosquitoes belonging to 12 species were sampled from May 2012 to April 2013, the equivalent of one wet season and one dry season (Table 4). Among mosquitoes collected, 673 specimens (69.67%) belonged to the *Anopheles* genus. Overall, the *Culicidae* fauna were very diversified in both rural (Sonsoro) and urban (Gansosso) sites. The mosquito collections were greater in rural Kandi (n = 668) compared to urban Kandi, which were 2.2 times less.

**Table 4:** Distribution of mosquito species caught from human baits in rural (Sonsoro) and urban (Gansosso) from May 2012 to March 2013

	**Study sites in Kandi**		
**Mosquito species**	**Rural**	**Urban**	**Total**
	**Sonsoro**	**Gansosso**	
*Anopheles gambiae*	628	39	667
*Anopheles funestus*	1	1	2
*Anopheles pharoensis*	1	1	2
*Anopheles coustani*	2	0	2
Non-anopheline	36	257	293
TOTAL	668	298	966

Furthermore, the predominant species of mosquito were not the same in the two locations. *An. gambiae* was the main species sampled in the rural area (Sonsoro), representing 94% of mosquitoes collected compared to 13.09% in Gansosso urban area. We noted a significant gap between the two locations, which could probably be justified by the ecology and specifically by the presence of a permanent waterway in the rural location (Sonsoro). The predominance of *An. gambiae* in rural Kandi was probably related to the availability of a suitable surface of water for the oviposition of this species.

#### ***Seasonal variation of the aggressive density of An. gambiae in rural and urban Kandi***

Table 5 details the total number of *An. gambiae* caught during the wet season (May-12 to S) and the dry season (November-12 to April-13) for each study site. Overall, the biting behavior of *An. gambiae* during the wet season was not the same in the two locations. In this period of the year, the aggressiveness of *An. gambiae* was mainly expressed on the inside of dwellings in rural Kandi (14.54 bites/night; p = 0.001) but on the outside in urban Kandi (1.08 bites/night; p = 0.008) (Table 5). The situation in urban Kandi could be explained by the widespread use of long-lasting insecticidal nets (LLINs) in times of abundance of mosquitoes (wet season) with the excito-repellency effect and the high exophily they induced. Conversely in the dry season, the observations were similar indoors and outdoors in both study sites (p >0.05). This could be justified by the change in the behavior of the populations which consists of sleeping outdoors because of the high night temperatures during the dry season.

**Table 5:** **Seasonal variation in the density of *An. gambiae *** **inside and outside of dwellingsin rural (Sonsoro) and urban (Gansosso) from May 2012 to March 2013**

		**Wet season (May-12 to October-12)**				**Dry season (November-12 to April-13)**				**Total**			
**Locations**		**N**	**Man night**	**HBR/night**	**p-value**	**N**	**Man night**	**HBR/night**	**p-value**	**N**	**Man night**	**HBR/night**	**p-value**
Rural Kandi	Indoor	349	24	14.54	0.001	2	32	0.06	0.065	351	56	6.27	0.003
(Sonsoro)	Outdoor	269	24	11.21		8	32	0.25		277	56	4.95	
Urban Kandi	Indoor	10	24	0.42	0.008	0	32	0	0.125	12	56	0.21	0.017
(Gansosso)	Outdoor	26	24	1.08		3	32	0.09		27	56	0.48	

N: number of specimens of *An. gambiae* caught.

HBR/night: human biting rate per human per night.

p-value: probability value.

#### ***Seasonal variation of the sporozoitic index in rural and urban Kandi***

Table 6 displays the variation of the *P. falciparum* CSP positivity rate in each season (wet versus dry) and in each study site (urban versus dry). A total of 286 specimens of *An. gambiae* were analyzed of which 9 were positive, representing an average sporozoitic index of 3.15%. In rural Kandi, we observed an increase of the sporozoitic index during the dry season probably due to the lower number of 10 vector specimens analysed for this period. Conversely, no significant seasonal variation was observed between sporozoitic indexes in urban Kandi.

**Table 6:** Seasonal variation of sporozoitic index in rural (Sonsoro) and urban (Gansosso) Kandi from May 2012 to April 2013

**Locations**	**Parameters**	**Wet season**	**Dry season**	**Total**
		**(May-12 to October-13)**	**(November-12 to April-13)**	
Rural Kandi (Sonsoro)	N tested	237	10	247
	Positive	7	1	8
	S (%)	2.95^a^	10^b^	3.24^x^
	**IC-95%**	[1.44-5.97]	[2.15-16.84]	[1.92-6.34]
Urban Kandi (Gansosso)	N tested	36	3	39
	Positive	1	0	1
	S (%)	2.78^a^	0^a^	2.56^y^
	**IC-95%**	[0.49-14.17]	[0.00-0.56]	[0.45-13.18]

S: sporozoitic index in %.

CI: confidence interval.

^a,b,x,y^Values with the same superscript do not differ significantly.

#### ***Seasonal variation of the EIR in rural and urban Kandi***

For each season, the EIR was higher in rural Kandi (Sonsoro) than in urban Kandi (Gansosso) (Table 7). For instance, the EIR for the 6-month wet season (May to October 2012) was estimated to the mean value of 136.73 infected bites (ib) in Sonsoro against only 7.51 ib in Gansosso, about 18 times higher in rural Kandi than in urban Kandi.

**Table 7:** Seasonal variation of EIR by locality and area in rural (Sonsoro) and urban (Gansosso) Kandi from May 2012 to April 2013

**Locations**	**Parameters**	**Wet season**	**Dry season**	**Total**
		**(May-12 to October-12)**	**(November-12 to April-13)**	
Rural Kandi (Sonsoro)	N vectors	618	10	628
	Man night	24	32	56
	HBR/night	25.75	0.31	11.21
	HBR/period	4635	56.25	4035.60
	S (%)	2.95	10	3.24
	**EIR/period**	**136.73**^**a**^	**5.63**^**b**^	**130.75**^**x**^
Urban Kandi (Gansosso)	N vectors	36	3	39
	Man night	24	32	56
	HBR/night	1.5	0.09	0.70
	HBR/period	270	16.88	252
	S (%)	2.78	0	2.56
	**EIR/period**	**7.51**^**a**^	**0**^**a**^	**6.45**^**y**^

S: sporozoitic index in %.

HBR: Human Biting Rate.

EIR: Entomological Inoculating Rate.

CI: confidence interval.

^a,b,x,y^Values with the same superscript do not differ significantly.

Nevertheless, we observed a similar seasonal variation in both sites. From wet to dry season, we observed a drastic reduction of 96% and 100% in the EIR in Sonsoro and Gansosso respectively. Overall, for the whole study duration of one year, we noted a higher transmission of malaria in rural Kandi (130.75 ib/h/year) than in urban Kandi (6.45 ib/h/year).

#### ***Prevalence of P. falciparum in urban and rural areas during the dry season***

##### 

**Characteristics of the children enrolled** A total of 400 children were enrolled for the investigation on the prevalence of malaria during the dry season. In Gansosso, girls were represented more (53%), whereas in Sonsoro boys were the majority (54.5%) (Table 8). The average age was 5.42 years in Gansosso and 6.44 years in Sonsoro (Table 3). Mean weights were similar in Sonsoro and Gansosso.

**Table 8:** Distribution of children according to the gender and the age

		**Urban area**			**Rural area**		
		**(Gansosso)**			**(Sonsoro)**		
**Qualitative**	**Modalities**	**N**	**%**	**CI-95%**	**N**	**%**	**CI-95%**
**Sexe**	Girls	106	53.00	[46.09-59.80]	091	45.50	[38.75-52.42]
	Boys	094	47.00	[40.20-53.91]	109	54.50	[47.58-61.25]
**Educational level**	Kindergarten	000	00.00	[00.00-01.88]	020	10.00	[06.57-14.94]
	1^st^level	199	99.50	[97.22-99.91]	058	29.00	[23.15-35.64]
	2^nd^level	001	00.50	[00.09-02.78]	096	48.00	[41.18-54.90]
	3^rd^level	000	00.00	[00.00-01.88]	026	13.00	[09.03-18.37]
**Quantitative**		**Average**	**CI-95**%	**Min/max**	**Average**	**CI-95**%	**Min/max**
**Age (years)**		05.42^a^	[05.30-05.53]	03.00/07.00	06.44^b^	[06.25-06.63]	03.00/09.00
**Weight (kg)**		19.54^a^	[19.16-19.92]	05.00/31.00	19.24^a^	[18.72-19.77]	10.00/29.00

^a,b^For a same parameter, values with the same superscript do not differ significantly.

##### Prevalence of *P. falciparum* in urban and rural Kandi

Positive RDTs were significantly higher in Sonsoro (41%) than in Gansosso (7.5%). In Sonsoro, the rate of positive RDTs was 5.47 times greater than that of Gansosso. Furthermore, we found 1 case of an invalid test in Gansosso and 2 in Sonsoro (Table 9).

**Table 9:** Results of the RDTs in schoolchildren in urban and rural Kandi

	**Urban area (Gansosso)**			**Rural area (Sonsoro)**		
**RDT results**	**N**	**%**	**IC-95%**	**N**	**%**	**IC-95%**
Invalid	001	00.50	[00.09-02.78]	002	01.00	[00.27-03.57]
Negative	184	92.00	[87.40-95.02]	116	58.00	[51.07-64.63]
Positive	015	07.50	[04.60-12.00]	082	41.00	[34.42-47.92]
Total	200	100.00	-	200	100.00	-

##### 

**Influences of collateral factors on the prevalence of malaria** A logistic regression performed showed that only urbanization seems to have a significant influence on the incidence of malaria (LR-test, p = 0.00000041). Overall and theoretically, it appears that for a child living in Sonsoro, the risk of malaria in the dry season is increased by 8 times compared to a child living in Gansosso of the same profile. Gender, level of education, age and weight did not seem to play any important role in the risk of malaria (Table 10).

**Table 10:** Logistic regression evaluating the influence of urbanization, gender, educational level, age and weight of children on the incidence of malaria in the commune of Kandi

**Factors**	**Modalities**	**N (%)**	**Coefficients**	**OR.adj**^**1**^	**IC-95%**^**2 **^**(OR.adj)**	**p (Wald test**^**3**^**)**	**p (LR-test**^**4**^**)**
**Areas**	Urban	15(07.61)	-	01.00	-	-	0.00000041
	Rural	82(41.62)	02.06	07.88	[03.54-17.51]	0.00000041	
**Gender**	Girls	41(21.13)	-	01.00	-	-	0.613
	Boys	56(27.59)	00.13	01.14	[00.69-01.90]	0.613	
**Educational level**	Kindergarten	06(31.58)	-	01.00	-	-	0.32
	1^st^level	38(14.84)	-00.38	00.68	[00.19-02.44]	0.555	
	2^nd^level	51(47.42)	00.20	01.95	[00.68-05.56]	0.549	
	3^rd^level	07(28.00)	-01.30	00.27	[00.04-01.67]	0.156	
**Age**			00.20	01.22	[00.81-01.85]	0.339	0.339
**Weight**			00.04	01.04	[00.94-01.16]	0.444	0.444

^1^adjusted Odds ratios.

^2^95% Confidence Interval of the adjusted Odds ratios.

^3^p-value for Wald test.

^4^p-value for LR test.

## Discussion

We observed that the entomological profile was not the same in rural and urban Kandi. *An. gambiae*, the main malaria vector in Africa is very abundant in rural settings of Kandi as compared to that in rural areas. This result suggested that there is an ecological dichotomy between the two study sites, the rural (Sonsoro) and the urban (Gansosso). Moreover, it is well known that urbanization greatly influenced the *Culicidae* diversity and is antagonistic to the anopheline abundance. The situation in drought was characterized by a significant decrease in vector density. In view of larval sampling data, we think that the few anopheles caught during the dry season, especially in urban Kandi, come from household water sources (canisters, jars) which become potential breeding sites in this period of the year. The situation was similar in rural Kandi although the proximity to a permanent water site appeared to be a risk factor. The poor conservation of water stocks in households in a period of scarcity of classical breeding sites indirectly causes the domestication of mosquito larval habitats. This could be avoided if the receptacles containing water supplies are well covered regularly.

Our results showed a lower aggressive density of *An. gambiae* during drought periods in both rural and urban settings as compared to the values in the wet season, nevertheless the HBR for the semester drought was 3.4 times higher in rural Kandi than in urban Kandi (p < 0.001). It is thought that the low density and the low aggressiveness often noted for *An. gambiae* in the dry season is particularly due to the fact that *Anopheles* adults are scattered in shelters for a summer diapause (aestivation), to ensure its survival [15]. Thus, sampling them is very difficult because, on one hand, they are resting in hiding, and on the other hand, they reduce the frequency of blood meals [26] as having significantly slowed the rate of ovarian development [27].

Similarly, we also observed significant seasonal variation in the infectivity of *An. gambiae* to *P. falciparum* in both study sites. The EIR was nil in urban Kandi due to the fact that no anopheline vector was found to be positive for the CSP of *P. falciparum.* The very low number of only 5 *An. gambiae* captured in urban Kandi during the whole drought period could explain it. For the whole study duration, we noted a higher transmission of malaria in rural Kandi (130.75 ib/h/year) than in urban Kandi (6.45 ib/h/year). The level of malaria transmission in rural and urban Kandi is more of a concern than in some locations under a wet climate in southern Benin. There are for instance Ossomou in the southeastern part (0 ib per semester) [28] and Ouidah-Kpomassè-Tori health district in the southwest (0.7 ib per 100 night) [29].In addition, the epidemiological situation of malaria is more complicated in Kandi than in many locations in Sub-Saharan Africa such as Bobo-Dioulassou in Burkina Faso [30], Edea in Cameroon [31], Dassilami in Gambia [32], Dakar (around Grande Niaye marsh) in Senegal [33] where the annual EIRs were lower.

The entomological data in Kandiwas perfectly reflected in the results of the parasitological survey conducted in both locations. Our results showed that Sonsoro in rural Kandi is a location of very high malaria risk during periods of drought. The prevalence of *P. falciparum* (RDTs) results showed that malaria is much more acute in the rural area especially in the dry season. The absence of urbanization has been identified as an important collateral risk factor. Our findings corroborate those of Gardiner *et al.*[34] in southeastern Ghana, Watts *et al.*[35] in Zambia, Trape [36] in Brazzaville, and Hay *et al.*[37] in most parts of tropical Africa who have all noticed that the lower density of *Anopheles* accompanying urbanization leads to significant parasitological and clinical implications [34-37].

Moreover, the low frequency of both positive RDTs and clinical malaria cases observed in Gansosso could also be due to self-treatment in urban populations and home care management of malaria once the onset of symptoms such as fever occurs. According to Gardiner *et al.*[34], the low incidence of malaria in an urban community, especially children under 10 years old is associated with widespread use of anti-malarial drugs such as a prophylactic treatment. This is probably one of the reasons why we note a gap in urban and rural Kandi results [34].

The existence of malaria cases in the dry season in urban areas where mosquito larval habitats and *Anopheles* vectors were scarce illustrated perfectly the perennity and the endemicity of malaria transmission in the municipality of Kandi, as in Bénin. The drought malaria prevalence recorded in urban Kandi (7.5%) is still relatively high for areas outside Africa, such as Asian countries. According to Trape [38], there is a threshold level of transmission beyond which malaria cases become common to the point that the malaria infection truly becomes a problem. Thus, above 2 ib per individual per year in a location, malaria transmission should be of major concern [38]. The annual value of EIR in urban Kandi (6.45 ib/h) exceeded the “threshold of Trape” and easily explained the malaria prevalence recorded there.

To this end, the indoor residual spraying of insecticide will be ideal, especially when we consider the fact that in and around Kandi, there is a single period of malaria transmission (May to October). However, given the very high night temperatures during the dry season, which compels individuals to sleep outside, malaria transmission prevention during this season of the year could be strengthened outdoors. Thus, populations may be encouraged to sleep outdoors with their bednets. Meanwhile, it is clear that due to the excessive heat, the use of bed nets during the dry season will be very difficult. In such conditions, it is important to provide them with new tools adapted to their practices and habits. The hypothesis of repellent perfumes or deodorants holds a great potential in this regard.

Our study is not without some limitations. The prevalence of malaria can be more accurately estimated by the use of PCR based diagnostics or microscopy instead of RTD [39,40]. However, since the primary outcome of interest in this study was recent infection and a global view of malaria prevalence as opposed to parasitaemia, we believe this limitation will neither significantly affect interpretation of the results nor affect the scientific quality of the study. Another potential limitation of this study is the fact of not to present the larval density in the drought-refugia of mosquitoes. Our larval sampling data were qualitative in order to just identify the atypical habitats which maintain mosquito breeding and ipso facto malaria transmission when classical larval habitats have dried up.

## Conclusion

Our results showed a high level of malaria transmission in the municipality of Kandi. Household water source plays an important role in maintaining the breeding of Anopheles larvae and the malaria transmission in Kandi. In rural settings, the proximity to a permanent water site could probably be the aggravating factor. These were the causes of the several malaria cases often recorded in Sonsoro during periods of drought. However, both entomological and parasitological data suggested that the epidemiological issue of malaria is not uniform in Kandi, especially in drought periods. At Sonsoro in the peripheral zone, the prevalence of malaria during the dry season was eight times higher than at Gansosso in downtown Kandi. Among the likely factors involved, the level of urbanization has been identified as potentially having a significant influence on the incidence of malaria in the municipality of Kandi.

## Competing interests

The authors declare that they have no competing interests.

## Authors’ contributions

RG and MA designed the study. RG, VG, RFA, AS and FOA carried out the field activities. RG and FOA analyzed the data. RG drafted the manuscript. MA, RA and RO critically revised the manuscript for intellectual content. All authors read and approved the final manuscript.
